# Advancements in Tuberculosis Diagnostics: A Comprehensive Review of the Critical Role and Future Prospects of Xpert MTB/RIF Ultra Technology

**DOI:** 10.7759/cureus.57311

**Published:** 2024-03-31

**Authors:** Sankalp Yadav, Gautam Rawal, Madhan Jeyaraman, Naveen Jeyaraman

**Affiliations:** 1 Medicine, Shri Madan Lal Khurana Chest Clinic, New Delhi, IND; 2 Respiratory Medical Critical Care, Max Super Speciality Hospital, New Delhi, IND; 3 Clinical Research, Viriginia Tech India, Dr. MGR Educational and Research Institute, Chennai, IND; 4 Orthopaedics, ACS Medical College and Hospital, Dr. MGR Educational and Research Institute, Chennai, IND

**Keywords:** bacterial load, cbnaat/ xpert/ rif assay, mtb (mycobacterium tuberculosis), tuberculosis, xpert mtb/rif ultra technology

## Abstract

Tuberculosis remains a persistent global health challenge, demanding swift and accurate diagnostic methods for effective treatment. The emergence of the Xpert MTB/RIF Ultra system marks a significant milestone in combating tuberculosis, streamlining the identification of *Mycobacterium tuberculosis*, and advancing our pursuit of eradicating the disease.

Delving into the therapeutic landscape of tuberculosis and rifampicin resistance, this scientific narrative review offers a comprehensive exploration. It begins by delving into the historical backdrop and the hurdles encountered with traditional tuberculosis diagnostics. From there, it traces the journey of the Xpert MTB/RIF technology, underscoring its molecular underpinnings.

In this narrative review, the performance of the Xpert MTB/RIF Ultra system undergoes thorough scrutiny, encompassing investigations into sensitivity, specificity, and comparisons with alternative diagnostic methods. The spotlight shines on its clinical applications across diverse scenarios, from diagnosing pulmonary and extrapulmonary tuberculosis to its pivotal role in identifying rifampicin resistance. The study also evaluates its clinical efficacy in enhancing patient outcomes and supporting global tuberculosis control initiatives.

However, the review does not shy away from discussing the challenges and limitations associated with the Xpert MTB/RIF Ultra system. It meticulously addresses concerns regarding cost, infrastructure requirements, and potential diagnostic inaccuracies.

Offering a panoramic view, the review assesses the system's impact in resource-constrained settings and its potential to bolster tuberculosis elimination endeavors worldwide. Peering into the future, it explores ongoing research avenues and potential enhancements in Xpert MTB/RIF Ultra technology, envisioning a landscape of improved performance, broader applications, and emerging diagnostic innovations in the realm of tuberculosis diagnostics.

## Introduction and background

Tuberculosis remains a persistent global health threat, exerting a significant toll on populations and healthcare systems worldwide [1]. With its profound impact on morbidity and mortality, tuberculosis necessitates advanced diagnostic tools that can expedite accurate identification, allowing for prompt treatment and containment [2]. In this manuscript, an overview of tuberculosis is presented, emphasizing its prevalence and challenges in diagnosis, and the critical need for innovative solutions is highlighted. Additionally, it introduces the Xpert MTB/RIF Ultra system, highlighting its significance in transforming tuberculosis diagnostics and improving patient outcomes.

Overview of tuberculosis and its global impact

For generations, the bacterium *Mycobacterium tuberculosis*, which causes tuberculosis, has been an important issue for public health. Millions of people continue to suffer from tuberculosis despite tremendous advancements in medical research, particularly in areas with inadequate resources. Though it can occasionally show up in other organs, the disease typically impacts the lungs, resulting in a variety of clinical manifestations [3].

Tuberculosis's global impact is staggering, with approximately 10 million new cases reported annually and an estimated 1.5 million deaths attributed to the disease (the incidence and prevalence of tuberculosis in India are 188 and 312 per one lakh [0.1 million] population, respectively) [1]. Factors such as poverty, overcrowding, and compromised immune systems contribute to its persistence, making it a major challenge for public health interventions. The rise of drug-resistant strains further complicates tuberculosis management, necessitating rapid and accurate diagnostic tools to curb transmission and improve treatment outcomes [4].

Introduction to Xpert MTB/RIF Ultra and its significance in tuberculosis diagnosis

The development of Xpert MTB/RIF Ultra represents a significant breakthrough in the diagnosis of tuberculosis. This molecular diagnostic platform, created by Cepheid, improves the efficacy of its forerunner, Xpert MTB/RIF, by increasing sensitivity and specificity in concurrently detecting rifampicin resistance and *M. tuberculosis* [5].

The significance of Xpert MTB/RIF Ultra lies in its ability to provide rapid and accurate results within a few hours, overcoming the limitations of traditional methods that often take weeks for conclusive outcomes. The technology relies on nucleic acid amplification techniques, specifically targeting the IS1081 and IS6110 as target regions of the *M. tuberculosis* genome and the rifampicin resistance-determining region of the rpoB gene. This dual-target approach not only enhances diagnostic accuracy but also facilitates early detection of drug-resistant strains, a crucial aspect in tailoring effective treatment regimens [5,6].

Beyond just its diagnostic capabilities, the release of Xpert MTB/RIF Ultra has significant ramifications. Because of its user-friendly design, it can be used in a variety of healthcare settings, including places with minimal resources, where tuberculosis is frequently most prevalent. This means that this technology could help with the global efforts to control and eradicate tuberculosis by speeding up the detection of patients, starting treatment on time, and so on [7].

Background 

Historical Context of Tuberculosis Diagnostics

The historical trajectory of tuberculosis diagnostics reflects a longstanding struggle to effectively identify and combat this infectious disease. Tuberculosis has plagued humanity for millennia, leaving an indelible mark on societies and necessitating ongoing innovation in diagnostic approaches [8].

Early attempts to diagnose tuberculosis were often rudimentary, relying on clinical observation of symptoms such as cough, weight loss, and hemoptysis. The iconic association of tuberculosis with lung cavities led to the development of chest radiography in the early 20th century, providing a visual means to detect pulmonary involvement. However, these methods lacked specificity and were unable to distinguish tuberculosis from other respiratory conditions [9].

The introduction of acid-fast staining in the late 19th century, notably by Robert Koch, marked a significant breakthrough. This technique allowed the identification of *M. tuberculosis* in sputum samples, providing a more direct and specific means of diagnosis. Nonetheless, acid-fast staining was limited by its sensitivity and often failed to detect low bacterial loads, particularly in extrapulmonary tuberculosis [10].

Despite these advancements, the need for more reliable and rapid diagnostic tools became increasingly evident, particularly with the emergence of drug-resistant tuberculosis strains. This realization prompted a shift towards molecular diagnostics, leading to the development of the Xpert MTB/RIF Ultra system, a groundbreaking technology designed to address the limitations of traditional methods [11].

Introduction to Traditional Methods of Tuberculosis Detection and Challenges Associated With Them

Traditional methods of tuberculosis detection, including sputum smear microscopy and culture, played pivotal roles in shaping diagnostic practices. Sputum smear microscopy, based on acid-fast staining, became a cornerstone in tuberculosis diagnosis during the mid-20th century. However, its sensitivity proved suboptimal, especially in cases of paucibacillary or extrapulmonary tuberculosis, limiting its effectiveness [12].

Culturing *M. tuberculosis*, though highly specific, was hindered by its prolonged turnaround time, often several weeks. This delay impeded the timely initiation of treatment, allowing for the continued spread of the disease. Moreover, culture-based methods were resource-intensive, requiring specialized facilities, skilled personnel, and a controlled environment [13].

Challenges associated with traditional methods extend beyond sensitivity and turnaround time. In resource-limited settings, the infrastructure necessary for sophisticated laboratory techniques is often lacking, hindering the widespread implementation of these diagnostic approaches [14]. Additionally, the reliance on empirical treatment in the absence of rapid and accurate diagnostic tools could result in the emergence of drug-resistant tuberculosis strains, escalating the complexity of tuberculosis management.

As the limitations of traditional methods became increasingly apparent, there arose a critical need for a diagnostic paradigm shift. These challenges opened avenues for newer diagnostic tests like the Xpert MTB/RIF Ultra system.

## Review

Development of Xpert MTB/RIF Ultra

Historical Evolution of the Xpert MTB/RIF Technology

The introduction of the Xpert MTB/RIF technology represents a paradigm shift in the landscape of tuberculosis diagnostics, marked by a commitment to enhancing speed, accuracy, and accessibility. The roots of this transformative technology trace back to the early 2000s, when molecular diagnostics emerged as a promising avenue for overcoming the limitations of traditional tuberculosis detection methods [15].

The creation of the Xpert MTB/RIF test laid the groundwork for the Xpert MTB/RIF technology. This first version was first presented by Cepheid in 2010 and used real-time polymerase chain reaction to detect rifampicin resistance and amplify the deoxyribonucleic acid (DNA) of *M. tuberculosis*. The rifampicin resistance-determining region of the rpoB gene was the area that was targeted for rifampicin resistance detection [16].

Xpert MTB/RIF quickly gained recognition for its revolutionary impact on tuberculosis diagnostics. By significantly reducing turnaround times, it addressed the critical need for rapid identification of tuberculosis cases, allowing for prompt initiation of treatment. Its automated nature, coupled with the ability to simultaneously identify rifampicin resistance, provided a comprehensive diagnostic solution [15].

Key Improvements Leading to the Development of Xpert MTB/RIF Ultra

Xpert MTB/RIF Ultra was developed in response to the success of Xpert MTB/RIF, with the goal of improving and enhancing the capabilities of tuberculosis diagnosis. Through this iterative approach, a number of significant advancements were made that together improved the technology's sensitivity, specificity, and adaptability.

Enhanced sensitivity: The primer and probe designs made possible by Xpert MTB/RIF Ultra allowed for more effective target DNA amplification. A major drawback of the previous version was addressed by the increased sensitivity, which made it possible to identify lower bacterial loads, especially in cases with paucibacillary or extrapulmonary tuberculosis [17].

Additional molecular targets: The *M. tuberculosis* genome's IS6110 region is one of the additional molecular targets that were added to improve Xpert MTB/RIF Ultra's diagnostic accuracy. The assay's robustness was enhanced by the multi-target strategy, which also decreased the possibility of false-negative results and helped to detect tuberculosis more thoroughly [18].

Increased detection of rifampicin resistance: By focusing on more rpoB gene areas, Xpert MTB/RIF Ultra increased the scope of its rifampicin resistance coverage. The objective of this modification was to increase the assay's capacity to detect a variety of rifampicin-resistant bacteria by capturing a wider range of resistance mutations [17].

User-friendly design: Xpert MTB/RIF Ultra kept the user-friendly design of its predecessor, acknowledging the necessity for broad applicability. Even in environments with inadequate laboratory equipment and technological know-how, the system was nevertheless able to provide automated sample processing and result interpretation [7].

The continuous enhancements that culminated in the creation of Xpert MTB/RIF Ultra have jointly led to its standing as the gold standard in the diagnosis of tuberculosis.

Principle of operation

The Xpert MTB/RIF Ultra system is based on advanced molecular concepts and uses rifampicin resistance and *M. tuberculosis* detection through nucleic acid amplification techniques. The technique ensures quick and precise findings by combining real-time detection with a polymerase chain reaction [15]. The molecular mechanisms underlying Xpert MTB/RIF Ultra are explained in full below.

Nucleic acid extraction: First, DNA and other nucleic acids are extracted from the clinical sample (often sputum). In order to collect the desired genetic material for amplification and detection later on, this step is essential [19].

Multiplex real-time polymerase chain reaction: The hemi-nested to fully nested polymerase chain reactions can be converted using Xpert MTB/RIF Ultra. Multiplex real-time polymerase chain reaction, a very specific and sensitive method, is used in this. Multiplexing allows the simultaneous amplification of multiple DNA targets in a single reaction, optimizing the diagnostic process [19].

Target regions for *M. tuberculosis* detection: The system targets specific DNA sequences within the genome of *M. tuberculosis*, i.e., multi-copy genes IS6110 and IS1081. One of the key regions is the IS6110 insertion element, a highly conserved segment present in multiple copies in the *M. tuberculosis* genome. The inclusion of the IS6110 region enhances the sensitivity of the assay, enabling the detection of paucibacillary loads [19].

Rifampicin resistance detection: Concurrently, Xpert MTB/RIF Ultra concentrates on finding mutations connected to resistance to rifampicin. Targeting different areas within the rifampicin resistance-determining region of the rpoB gene accomplishes this. Rifampicin resistance mutations are common in this region and are indicative of resistance to this critical first-line anti-tuberculosis drug [5].

Dual-target approach: Because it targets both *M. tuberculosis* detection and rifampicin resistance in a single assay, Xpert MTB/RIF Ultra's dual-target strategy is what gives it its potency. By amplifying and detecting multiple genetic markers simultaneously, the system provides a comprehensive diagnosis, significantly reducing the time required for results compared to traditional methods [5].

Real-time detection and analysis: Real-time polymerase chain reaction allows for continuous monitoring of the amplification process. Fluorescent probes specific to the target regions emit signals as the DNA is amplified. The real-time detection enables the system to quantify the amount of DNA present in the sample, providing information about the bacterial load [5].

Interpretation of results: The system interprets the data automatically; positive signals mean that *M. tuberculosis* and, if relevant, rifampicin resistance are present. Xpert MTB/RIF Ultra's automated features lower the possibility of human mistakes in result interpretation, improving the accuracy of the diagnostic result [5].

Performance evaluation

Summary of Studies Assessing the Sensitivity and Specificity of Xpert MTB/RIF Ultra

Xpert MTB/RIF Ultra's performance has been methodically assessed in a number of studies, which have examined its sensitivity and specificity in various clinical scenarios. All of the evidence points to how strong the system's diagnostics are.

Sensitivity: Research continuously shows that Xpert MTB/RIF Ultra has a high sensitivity for identifying *M. tuberculosis*. The assay's increased sensitivity is made possible by the inclusion of the IS6110 area, which makes it possible to identify cases of paucibacillary illness, extrapulmonary tuberculosis, and low bacterial loads [20-22].

Specificity: Xpert MTB/RIF Ultra comes with a reduced specificity compared to Xpert MTB/RIF; however, it accurately discriminates between *M. tuberculosis* and non-tuberculosis pathogens. The targeted approach to rifampicin resistance mutations ensures specificity in identifying drug-resistant strains, which is crucial for guiding appropriate treatment strategies [20-22].

Performance in rifampicin resistance detection: One of the system's main advantages is its capacity to identify mutations that provide rifampicin resistance. Research continuously shows that the rifampicin resistance-determining region of the rpoB gene exhibits significant sensitivity in detecting these mutations. This is particularly relevant for initiating timely treatment with appropriate second-line drugs in cases of drug-resistant tuberculosis [20-22].

Impact on patient management: Research has looked at the clinical effects of Xpert MTB/RIF Ultra on patient care in addition to analytical performance. This system's ability to diagnose quickly and accurately results in prompt treatment initiation, lowering the risk of transmission, and enhancing patient outcomes [16].

Comparison With Other tuberculosis Diagnostic Methods, Particularly the Earlier Version of Xpert MTB/RIF

Improved sensitivity: Studies comparing Xpert MTB/RIF Ultra to its predecessor show a continuous improvement in sensitivity. The improved performance is ascribed to the incorporation of supplementary target regions, particularly the IS6110 element, which tackles constraints related to extrapulmonary and paucibacillary tuberculosis [20-22].

Enhanced detection in HIV-positive individuals: Xpert MTB/RIF Ultra shows superior sensitivity in HIV-positive individuals, a population known for increased susceptibility to tuberculosis. The improved sensitivity is crucial in settings with a high prevalence of HIV co-infection [23].

Specificity retention: Xpert MTB/RIF Ultra maintains strong specificity, ensuring reliable distinction between non-tuberculosis pathogens and *M. tuberculosis*, even with target regions expanding. This is crucial to prevent needless medical intervention and any problems [7].

Decreased rate of false-negative results: Comparative studies show that Xpert MTB/RIF Ultra has a lower rate of false-negative results, especially when there is a low bacterial load. This is explained by the multi-target approach's increased sensitivity [7].

Detection of rifampicin resistance: In terms of rifampicin resistance detection, Xpert MTB/RIF Ultra continuously performs better than the previous version. The system's capacity to recognize various mutations linked to resistance is improved by the broader coverage of target regions [24].

Applications in tuberculosis diagnosis

Exploration of Various Clinical Settings

Xpert MTB/RIF Ultra has proven to be a flexible solution in a variety of clinical contexts, greatly assisting in the diagnosis of tuberculosis in a wide range of patient demographics. It is a useful instrument in the fight against tuberculosis because of its influence, which goes beyond typical diagnostic settings.

Primary healthcare facilities: Xpert MTB/RIF Ultra's user-friendly design and rapid results make it well-suited for deployment in primary healthcare facilities. Its simplicity allows healthcare providers with varying levels of expertise to administer the test, facilitating the early detection of tuberculosis cases in resource-limited settings [17].

HIV-positive individuals: In populations with a high prevalence of HIV, where tuberculosis incidence is elevated, Xpert MTB/RIF Ultra plays a crucial role. Its heightened sensitivity ensures accurate diagnosis in HIV-positive individuals, addressing the challenges posed by immunocompromised states and the increased risk of tuberculosis reactivation [23,25].

Pediatric tuberculosis diagnosis: Because pediatric tuberculosis is frequently paucibacillary in character and might be challenging to get sufficient sputum samples, it poses special obstacles. Because of its high sensitivity, particularly in extrapulmonary infections, Xpert MTB/RIF Ultra is a useful diagnostic test for tuberculosis in children, increasing the likelihood of prompt treatment and effective outcomes [26].

Tuberculous meningitis diagnosis: Due to the low bacillary load in cerebrospinal fluid (CSF) fluid, the diagnosis of tuberculous meningitis, a severe type of extrapulmonary tuberculosis, has historically been difficult. The capacity of Xpert MTB/RIF Ultra to identify low bacterial loads improves its usefulness in quickly detecting tuberculous meningitis, enabling rapid and suitable therapy [23].

Discussion on Its Role in Diagnosing Pulmonary and Extrapulmonary Tuberculosis

Pulmonary tuberculosis diagnosis: Xpert MTB/RIF Ultra is highly effective in diagnosing pulmonary tuberculosis. Its sensitivity in sputum samples is noteworthy, allowing for the rapid identification of *M. tuberculosis* even in cases with low bacillary loads. The ability to deliver results within a few hours expedites the initiation of treatment and helps curb further transmission [7].

Extrapulmonary tuberculosis diagnosis: Extrapulmonary tuberculosis, characterized by *M. tuberculosis* involvement outside the lungs, poses diagnostic challenges due to the varied clinical presentations and the paucibacillary nature of the disease. Xpert MTB/RIF Ultra's multi-target approach, including the IS6110 region, enhances its sensitivity in extrapulmonary cases. It has proven valuable in diagnosing tuberculosis affecting organs such as the lymph nodes, pleura, bones, and central nervous system [27].

Evaluation of Performance in Comparison to Traditional Drug Susceptibility Testing Methods

Comparison with phenotypic drug susceptibility testing (DST): The gold standard for DST has always been traditional phenotypic DST, which involves cultivating *M. tuberculosis* and measuring growth when medicines are present. When it comes to identifying rifampicin resistance, Xpert MTB/RIF Ultra exhibits similar sensitivity and specificity to phenotypic DST, with the added advantage of yielding data in a much shorter amount of time [11].

Advantages over conventional methods: Xpert MTB/RIF Ultra offers several advantages over conventional DST methods, including a reduced turnaround time, lower technical expertise requirements, and the ability to detect resistance directly from clinical specimens. The system's ability to function without the need for bacterial culture is especially valuable in settings with limited laboratory infrastructure [11].

Concordance with molecular DST techniques: Rifampicin resistance has been found using molecular DST techniques, including line probe tests [28]. The remarkable concordance that Xpert MTB/RIF Ultra exhibits with various molecular approaches validates its dependability and precision in detecting mutations that lead to rifampicin resistance.

Impact on global tuberculosis control: The global efforts to control tuberculosis have been significantly impacted by the widespread use of Xpert MTB/RIF Ultra [7]. The method helps to ensure the timely beginning of appropriate therapy, lowering the danger of continued transmission and the establishment of extensively drug-resistant tuberculosis by delivering fast and reliable results regarding rifampicin resistance.

Clinical utility

Review of the Impact on Clinical Decision-Making

With its revolutionary approach to quick and precise identification of *M. tuberculosis* and critical resistance profiles, Xpert MTB/RIF Ultra has completely redesigned clinical decision-making in the field of tuberculosis diagnosis. The system has an impact on many aspects of clinical decision-making, including the start of therapy, infection prevention strategies, and general patient care.

Timely treatment initiation: Fast-tracking the start of treatment is one of Xpert MTB/RIF Ultra's most important accomplishments. The rapid turnaround time of the system, providing results within a few hours, allows clinicians to promptly start appropriate anti-tuberculosis therapy [7]. This acceleration in treatment initiation is especially critical in preventing disease progression, reducing morbidity, and minimizing the risk of transmission within communities.

Identification of drug-resistant tuberculosis: Xpert MTB/RIF Ultra's influence on clinical decision-making is increased by its direct ability to identify rifampicin resistance from clinical specimens. Early detection of drug-resistant tuberculosis strains alerts medical professionals to the need for different drug regimens, reducing the usage of inefficient drugs and enhancing the effectiveness of treatment as a whole [29].

Impact on infection control measures: Xpert MTB/RIF Ultra's ability to provide a fast and accurate diagnosis has an impact on infection control measures. Healthcare facilities can rapidly implement adequate infection control policies by identifying cases of tuberculosis [29]. This lowers the risk of nosocomial transmission and safeguards patients and healthcare personnel.

Cutting down on diagnostic complications and delays: Xpert MTB/RIF Ultra's role in minimizing diagnostic delays is instrumental in preventing complications associated with delayed tuberculosis diagnosis [20]. Early identification of tuberculosis allows for timely intervention, reducing the severity of the disease, preventing the development of drug resistance, and ultimately improving overall patient outcomes [7].

Challenges and limitations of Xpert MTB/RIF Ultra

Challenges

Resource limitations: In settings with restricted resources, Xpert MTB/RIF Ultra implementation may present difficulties. In areas with few healthcare resources, the initial cost of purchasing the technology as well as continuing costs for consumables and maintenance might put a burden on budgets [30, 31].

Training needs: To utilize Xpert MTB/RIF Ultra efficiently, healthcare workers must get sufficient training. Users may experience a learning curve in environments with limited training opportunities, which could have an impact on test accuracy.

Maintenance and quality control: Reliable results depend on the Xpert MTB/RIF Ultra devices receiving correct maintenance and routine quality control. Situations where there is a lack of technical know-how or where it is difficult to get regular maintenance services could provide challenges.

Limitations

Cost: Xpert MTB/RIF Ultra cartridge and instrument costs can be prohibitive, especially in settings with limited resources. Widespread adoption may face obstacles due to the initial investment and continuing operation.

Infrastructure requirements: Like Xpert MTB/RIF, Xpert MTB/RIF Ultra relies on a stable power supply and controlled environmental conditions for optimal performance. In regions with unreliable electricity or limited laboratory infrastructure, maintaining the necessary conditions can be challenging [31].

False positives and false negatives: While Xpert MTB/RIF Ultra demonstrates high sensitivity and specificity, false positives and false negatives can still occur. False positives may be associated with contamination during sample collection or processing, while false negatives may arise in cases of low bacterial loads or mutations not covered by the assay [31].

Detection threshold and paucibacillary cases: The concentration of bacteria in the sample affects Xpert MTB/RIF Ultra's sensitivity. The specificity may drop in paucibacillary tuberculosis patients when the bacterial burden is low, which could have an impact on the outcome [5].

Limited drug resistance coverage: While Xpert MTB/RIF Ultra is effective in detecting rifampicin resistance, it does not provide comprehensive information about resistance to other anti-tuberculosis drugs. This limitation necessitates additional testing for a complete drug susceptibility profile. However, a few studies have suggested that the same sample could be used for the detection of further resistance [32].

Strain diversity: The effectiveness of Xpert MTB/RIF Ultra may be impacted by the genetic diversity of *M. tuberculosis* strains found throughout the world. Because unusual or novel mutations may not be discovered due to the assay's concentration on common mutations, there may be difficulties in diagnosing patients [33].

Limited capacity for strain differentiation: Xpert MTB/RIF Ultra does not distinguish between distinct strains; instead, it determines the presence of *M. tuberculosis* and rifampicin resistance. This restriction can be significant in epidemiological research or scenarios where strain monitoring is crucial.

Inability to detect non-tuberculous Mycobacteria (NTM): The second limitation of Xpert MTB/RIF Ultra is its specificity to *M. tuberculosis*; it is unable to identify NTM. The assay may not provide information on these clinically important pathogens in areas where NTM infections are common.

In order to maximize the utilization of Xpert MTB/RIF Ultra and take into account supplementary solutions to address particular operational and diagnostic demands, it is imperative to acknowledge these obstacles and limits. Future research and technological developments might help to lessen some of these restrictions.

Global Impact of Xpert MTB/RIF Ultra on tuberculosis control programs

Assessment of the Impact

Enhanced case detection: Worldwide, case detection rates have been considerably raised using Xpert MTB/RIF Ultra. Due to the use of multi-copy insertion elements (IS1081 and IS6110) as Mtb targets, it has a significantly lower limit of detection (LOD-15.6 CFU/ml) than Xpert (LOD-112.6 CFU/ml). It also yields an additional semi-quantitative "trace" result with an indeterminate rifampicin resistance and delivers the result in 77 minutes [17].

Its high sensitivity in detecting *M. tuberculosis*, especially in cases of extrapulmonary and paucibacillary tuberculosis, has led to more accurate and timely diagnoses [34]. This, in turn, has contributed to improved patient outcomes and reduced transmission rates.

Rapid identification of drug resistance: The system's ability to rapidly detect rifampicin resistance directly from clinical specimens has revolutionized tuberculosis control programs. Early identification of drug-resistant strains allows for prompt initiation of appropriate treatment, preventing the spread of drug-resistant tuberculosis and improving overall treatment success rates [17].

Impact on public health interventions: Xpert MTB/RIF Ultra has facilitated targeted public health interventions by providing timely and accurate diagnostic information. The system's rapid turnaround time has enabled healthcare authorities to implement infection control measures swiftly, reducing the risk of nosocomial transmission and protecting vulnerable populations [17,34].

Reduced diagnostic delays: By minimizing diagnostic delays associated with traditional methods, Xpert MTB/RIF Ultra has played a crucial role in preventing the progression of tuberculosis and its complications. Early diagnosis contributes to better patient outcomes and supports overall tuberculosis control efforts [33].

Role in Resource-Limited Settings

Increased accessibility in resource-limited settings: In areas with limited resources, Xpert MTB/RIF Ultra is now more widely available despite early financial difficulties. The technology is now more affordable thanks to initiatives by governments, non-governmental groups, and international organizations to negotiate lower prices or to subsidize it [15].

User-friendly design: Xpert MTB/RIF Ultra's user-friendly design makes it appropriate for deployment in environments with minimal technical know-how and laboratory infrastructure. Because of the automation of the system, a greater variety of healthcare facilities can utilize it because it depends less on highly qualified staff [15].

Potential for decentralized testing: The mobility and decentralized testing possibilities of Xpert MTB/RIF Ultra add to its appropriateness in environments with limited resources. Faster turnaround times are made possible by the capacity to perform tests closer to the point of care, which minimizes the requirement for sample transportation [33].

Contribution to Tuberculosis Elimination Efforts

Interrupting transmission chains: One of the most important functions of Xpert MTB/RIF Ultra is to provide fast and precise diagnosis, which is essential for breaking transmission chains. Early detection of tuberculosis cases, even those that are resistant to drugs, stops the disease from spreading throughout communities and medical facilities [35].

Supporting focused treatment plans: By spotting drug-resistant strains early in the diagnostic procedure, Xpert MTB/RIF Ultra helps to promote focused treatment plans. Clinicians can select the right drug regimens, maximize therapeutic efficacy, and stop the emergence of new drug resistance with the help of this information [36].

World Health Organization (WHO) Recommendations

The WHO gave a number of recommendations, as mentioned in Table 1 [31].

**Table 1:** World Health Organization recommendations for Xpert MTB/RIF Ultra DST: drug susceptibility testing, TB: tuberculosis [31]

Recommendations on Xpert MTB/RIF Ultra as initial tests in adults and children with signs and symptoms of pulmonary TB
Smear microscopy/culture and phenotypic DST should not be used as the initial diagnostic test for tuberculosis and rifampicin-resistant detection in sputum; instead, Xpert MTB/RIF Ultra should be used in adults with signs and symptoms of pulmonary TB and without a prior history of TB (≤5 years) or with a remote history of TB treatment (>5 years since end of treatment).
Instead of smear microscopy/culture and phenotypic DST, Xpert MTB/RIF Ultra may be used as an initial diagnostic test for TB and for rifampicin-resistance detection in sputum in people with signs and symptoms of pulmonary tuberculosis, as well as a prior history of tuberculosis and treatment termination within the last five years.
Xpert MTB/RIF Ultra, as opposed to smear microscopy/culture and phenotypic DST, should be used as the first diagnostic test for TB and identification of rifampicin resistance in sputum or nasopharyngeal aspirate in children with signs and symptoms of pulmonary tuberculosis.
Recommendations on Xpert MTB/RIF Ultra as initial tests in adults and children with signs and symptoms of extrapulmonary TB
Xpert MTB/RIF Ultra should be utilized in cerebrospinal fluid as a first diagnostic test for TB meningitis instead of smear microscopy/culture in adults and children exhibiting signs and symptoms of the disease.
Xpert MTB/RIF Ultra may be used in lymph node aspirate and lymph node biopsy as the initial diagnostic test instead of smear microscopy/culture in adults and children with signs and symptoms of extrapulmonary tuberculosis.
Instead of using culture and phenotypic DST, Xpert MTB/RIF or Xpert MTB/RIF Ultra should be used to detect rifampicin resistance in adults and children exhibiting signs and symptoms of extrapulmonary tuberculosis.
Recommendations on Xpert MTB/RIF Ultra repeated testing in adults and children with signs and symptoms of pulmonary TB
Retesting with Xpert MTB/RIF Ultra may not be necessary in persons with pulmonary tuberculosis symptoms and signs who tested positive for the test's trace positive result on the first attempt.
Xpert MTB/RIF Ultra testing in sputum or nasopharyngeal aspirate specimens may not be utilized in repeated testing for pulmonary tuberculosis in children exhibiting signs and symptoms of the disease in environments where the pretest probability is less than 5% and the original test result was negative.
Children with pulmonary tuberculosis signs and symptoms in conditions where the pretest likelihood is at least 5% and when the first Xpert MTB/RIF Ultra test yielded a negative result, may have a second Xpert Ultra test (making two tests total) using sputum and nasopharyngeal aspirate specimens.
Recommendations on Xpert MTB/RIF Ultra as initial tests for pulmonary TB in adults in the general population either with signs and symptoms of TB or chest radiograph with lung abnormalities or both
The initial test for pulmonary tuberculosis in adults in the general population may be replaced with the Xpert MTB/RIF Ultra in place of culture if they have signs and symptoms of tuberculosis or abnormalities in the lungs on a chest radiograph.
An initial Xpert MTB/RIF Ultra test instead of two Xpert MTB/RIF Ultra tests may be performed in persons in the general population who have either a positive TB symptom screen, a chest radiograph with lung abnormalities, or both.

Future directions of Xpert MTB/RIF Ultra technology

Ongoing Research

Improving sensitivity and specificity: Constant research endeavors to augment Xpert MTB/RIF Ultra's sensitivity and specificity. The performance of the assay can be enhanced by investigating additional genetic markers, refining the primer design, and utilizing cutting-edge molecular techniques, particularly in difficult cases like paucibacillary tuberculosis.

Strain distinction and genotyping: Adding features to the Xpert MTB/RIF Ultra platform for strain distinction and genotyping may be the main focus of future advancements. This could provide valuable information for epidemiological studies, outbreak investigations, and a deeper understanding of the genetic diversity of *M. tuberculosis*.

Multiplexed detection for drug resistance: Research efforts are exploring the feasibility of multiplexed detection for resistance to multiple anti-tuberculous drugs. This could increase the usefulness of Xpert MTB/RIF Ultra by giving clinicians guidance in choosing the most efficacious treatment plans through a more thorough medication susceptibility profile.

Point-of-care integration: More miniaturization and integration of Xpert MTB/RIF Ultra into point-of-care devices could result from technological advancements [36]. Decentralized testing would be made easier by this, allowing for quick diagnosis in situations where access to centralized facilities is restricted.

Improving epidemiological and surveillance research: The extensive usage of Xpert MTB/RIF Ultra may help improve epidemiological and surveillance research [37]. The consistent and dependable outcomes of the system offer important information for tracking trends in tuberculosis, comprehending the occurrence of medication resistance, and guiding policy choices.

Alignment with worldwide efforts: By emphasizing early detection and precise diagnosis, Xpert MTB/RIF Ultra is in line with worldwide tuberculosis elimination efforts. Its ability to lower tuberculosis morbidity and mortality contributes to larger initiatives aimed at achieving international goals for the disease's control and eradication [31].

Potential future developments in tuberculosis diagnostics

Next-generation sequencing (NGS): Next-generation sequencing holds promise as an emerging technology in tuberculosis diagnostics. NGS techniques can provide comprehensive genomic information, allowing for detailed strain characterization, the identification of resistance mutations, and a deeper understanding of the molecular epidemiology of tuberculosis [38].

Biosensors and nanotechnology: The development of biosensors and nanotechnology in tuberculosis diagnostics is an area of active research. These technologies aim to provide rapid, sensitive, and cost-effective detection of *M. tuberculosis *and resistance markers, potentially offering alternatives or complements to existing methods [39].

Host biomarkers and immune profiling: Research is underway to explore host biomarkers and immune profiling for tuberculosis diagnosis. Utilizing host responses and immune signatures may enhance diagnostic accuracy, particularly in cases where bacterial loads are low or in populations with a higher prevalence of extrapulmonary tuberculosis [40].

Integration of artificial intelligence (AI): Integration of artificial intelligence into tuberculosis diagnostics is a potential avenue for improving accuracy and efficiency. AI algorithms can assist in interpreting complex genetic data, optimizing result analysis, and identifying patterns that may contribute to more precise and personalized diagnostic approaches [41].

Whole-blood-based diagnostics: Innovations in whole-blood-based diagnostics are being explored to simplify sample collection and processing. These methods could offer advantages in resource-limited settings where obtaining sputum samples may be challenging [42].

Digital health solutions: The integration of digital health solutions, such as mobile applications and cloud-based platforms, could streamline the data management and reporting aspects of tuberculosis diagnostics. This could improve real-time surveillance, data sharing, and connectivity between diagnostic facilities [43,44].

Pilot programs for novel technologies: Pilot programs are likely to be initiated to assess the feasibility and effectiveness of novel tuberculosis diagnostic technologies in diverse settings. These programs aim to evaluate the real-world impact, scalability, and integration of emerging technologies into existing healthcare infrastructures [45].

## Conclusions

In essence, Xpert MTB/RIF Ultra has not only revolutionized tuberculosis diagnostics but has also become a cornerstone in the global fight against tuberculosis. Its valuable role in early and accurate diagnosis, especially in challenging cases, positions it as a key instrument in reshaping strategies for tuberculosis control and eventual elimination. As research and technological advancements continue, the trajectory of tuberculosis diagnostics is poised for further innovation, offering hope for more effective and comprehensive approaches to tackling this global health challenge.
